# Assessment of the Safety and Efficacy of a Raft-Forming Alginate Reflux Suppressant (Liquid Gaviscon) for the Treatment of Heartburn during Pregnancy

**DOI:** 10.5402/2012/481870

**Published:** 2012-11-04

**Authors:** Vicki Strugala, Julian Bassin, Valerie S. Swales, Stephen W. Lindow, Peter W. Dettmar, Edward C. M. Thomas

**Affiliations:** ^1^Technostics Limited, Daisy Building (2nd Floor), Castle Hill Hospital, Castle Road, Kingston Upon Hull HU16 5JQ, UK; ^2^Medical Centre, Netcare Linksfield Hospital, Suite 205, 12th Avenue, Linksfield West, Johannesburg 2037, South Africa; ^3^Elm Lane Surgery, Sheffield S5 7TW, UK; ^4^Department of Obstetrics & Gynaecology, Hull Royal Infirmary, Anlaby Road, Kingston Upon Hull HU3 2JZ, UK; ^5^Global Professional Relations, Reckitt Benckiser Group plc, 103-105 Bath Road, Slough SL1 3UH, UK

## Abstract

Gastro-oesophageal reflux (GER) and the symptoms of heartburn and regurgitation are common in pregnancy. These symptoms are transient and mostly resolve postpartum but have a negative impact on quality of life. Here, we present a prospective clinical evaluation of the safety and efficacy of an alginate raft-forming oral suspension that is licensed for use in pregnancy. The study was a multicentre, prospective, open-label, and baseline-controlled study of Liquid Gaviscon (LG) in the treatment of heartburn in pregnant women with current symptoms of heartburn and/or reflux requiring treatment (recruited 144). The efficacy of the study medication was rated by the investigator (primary endpoint) and patient. Treatment was deemed to be a success in 91% of patients as judged by the investigator (95% CI 85.0–95.3) and 90% (95% CI 84.1–94.8) when assessed by the patient themselves. Very few adverse events or serious adverse events were reported that were considered to be related to the study medication, and these were consistent with the normal population incidences. Serum sodium levels remained unchanged. This prospective open-label study in a large number of pregnant women has shown that LG is both safe and highly efficacious in the treatment of heartburn and GER symptoms in pregnancy.

## 1. Introduction

The symptoms of gastro-oesophageal reflux (GER), heartburn and regurgitation, are common in pregnancy with 40–80% suffering at some time during the pregnancy [1–4], and these symptoms can have a marked impact upon quality of life [2]. Although the prevalence of GER increases with advancing gestation time, a recent study has shown that the incidence of frequent GER (women starting with new symptoms) is consistent across the three trimesters (about 25% per trimester). Symptoms often begin in the latter part of the first trimester and persist into the second and then become more frequent and more severe in the third trimester. Often, symptoms are particularly prevalent postprandially and at night. 

Reflux appears to be a normal consequence of pregnancy which resolves postpartum. Complications are rare [4], but two studies now suggest that GER during pregnancy can predispose to GER later and thus may not be so innocuous [3, 5]. Rey et al. [3] found that in Spain 4.7% of women reported frequent GER symptoms 1 year postpartum compared to 1.3% of matched controls. Bor et al. [5] showed that in Turkey heartburn in pregnancy increased the risk of having heartburn 1 year later and that the risk increased with the number of births (baseline 6.4%, 1 delivery 17.7%, 2 deliveries 36.1%). 

Treatment of GER in pregnancy obviously needs to be conservative, and a step-up approach is advocated. Initially lifestyle modifications should be promoted (e.g., avoiding eating late at night and eating smaller meals). When medication is required the first port of call could be alginates or antacids because of their nonsystemic effects.

Alginate-based reflux suppressants such as Liquid Gaviscon and Gaviscon Advance (Reckitt Benckiser Healthcare (UK) Ltd) are licensed for use by pregnant women to combat the frequent symptoms of heartburn and regurgitation. Due to the physical mode of action and long-term experience, these products are shown to be safe to use in the high risk pregnancy and lactation population. However, due to the complicated ethical issues relating to evaluating medicines in pregnant women, clinical studies are few and far between and seldom have a placebo-control arm.

Lindow et al. [6] performed a formal safety and efficacy study using double strength product Gaviscon Advance (open label and uncontrolled) to treat symptoms of heartburn and regurgitation in pregnant women (*n* = 146). Efficacy of the treatment was deemed very good or good by 90% of the women with symptom relief usually within 10 minutes of taking the medication. Frequency and severity of heartburn decreased both in the day and at night after treatment.

A few older studies with small numbers have shown efficacy and safety of Gaviscon in the treatment of GER in pregnancy. Hutt et al. [7] carried out a drug-monitoring study of 52 women who were pregnant and had taken Gaviscon. 98.1% considered the treatment effective, and it was well or satisfactorily tolerated by all women. An Italian study by De Bellis et al. [8] evaluated Liquid Gaviscon suspension in 18 pregnant women during the second and third trimesters. All patients had effective control of symptoms on average within 10–15 days of commencing treatment. The medication was well tolerated with no signs of hypernatraemia or other adverse reactions. A French open-label trial [9] followed 50 pregnant women with reflux in the second and third trimesters treated with Gaviscon. All symptoms were significantly improved, including frequency, intensity, and duration of reflux symptoms with 98% positive efficacy. Tolerance of the medication was excellent and satisfactorily accepted with no evidence of hypertension or oedema. 

Due to the large number of pregnant users of the alginate-based reflux suppressant Liquid Gaviscon, there is a need for a robust safety and efficacy study of this product in pregnancy using the UK manufactured formulation that is marketed worldwide. Here, we present clinical data from a multicentre open-label study in pregnant women who took the medication for at least 4 weeks with the impact of the medication followed up until delivery of the baby. 

## 2. Methods

This was a multicentre, prospective, and open-label study of Liquid Gaviscon (LG) in the treatment of heartburn in pregnant women. There were eight centres involved in the study taken from general practice, hospital antenatal clinics or private clinics of which four were in the UK (all general practice centres) and four in South Africa (hospital ante-natal clinics, or private practice clinics). 

Pregnant women (≤38 weeks gestation) between the ages of 18 and 40 with current symptoms of heartburn and/or reflux requiring treatment, as a result of their current pregnancy, were eligible for the study. At the screening visit if the patient had experienced heartburn within the previous 72 hours, they were invited to participate in the study and written informed consent was obtained. 

Treatment was with Liquid Gaviscon oral suspension (Reckitt Benckiser Healthcare (UK) Ltd, Hull, UK) at a dose of 10–20 mL as required to relieve symptoms (prn), to a maximum of 80 mL per day, for 4 weeks. Treatment was allowed to be continued beyond 4 weeks if wished by the patient and deemed appropriate by the medical team.

Exclusion criteria for this study were known gastrointestinal (GI) disorders, and those with ischaemic heart disease, current uncontrolled clinically significant CNS, GI, metabolic, cardiac, hepatic dysfunction, renal dysfunction or systemic disease, or a condition which the investigator thought would compromise patient safety or interfere with efficacy assessment were excluded. With respect to the test product, patients with hypophosphatemia, phenylketonuria or those requiring a low sodium diet were excluded as were those with known sensitivity to any of the ingredients of LG or if the patient was routinely taking the product.

There were two study-specific visits (screening visit and assessment visit at 4 weeks)-but the women were also assessed at each routine antenatal visit, and postpartum. At the study assessment visit study medication use was recorded and frequency and severity of symptoms over the previous 72 hours were detailed. The investigator and patient rated the efficacy of the study medication (very good, good, acceptable, poor, and very poor). Any adverse events were recorded along with concurrent conditions, concomitant medication, and the results of blood pressure and blood (serum potassium and sodium) and urine tests. 

At the postpartum visit, the number of babies delivered was recorded along with gestational age at birth (premature, full term, and late delivery), delivery method, and AGPAR score (nominally at 1 and 5 minutes after birth).

Data analysis covered the safety population (all patients who had received at least one dose of medication), intention to treat (ITT) population (all patients who had received at least one dose of medication and had recorded efficacy data), and efficacy evaluable (EE) population (patients who adhered to the protocol). The primary endpoint of investigator assessment of treatment efficacy was summarised as frequency distribution with exact 95% confidence intervals (CI) for treatment success rate.

The study was conducted in accordance with the declaration of Helsinki and the ICH note for guidance on good clinical practice. Approval from appropriate independent ethics committees was obtained. The UK Medicines Control Agency was notified of the study under the clinical trials on marketed products scheme and similarly to the South African Medicines Control Council. This study was conducted using a marketed drug and performed under conditions of the product license.

## 3. Results

### 3.1. Patient Demography

The study recruited 144 patients of which 54 were in the UK and 90 were in South Africa. The study assessment visit was attended by 131 patients (91%), and postpartum data was available for 130 patients (90%).

The safety population comprised 142 patients (2 patients never took any medication), ITT 135 patients, and the EE 97 patients. Unless otherwise stated, the efficacy data presented in this paper is from the ITT population.

The mean (SD) age of the patients was 28.9 (5.4) years, and the majority were Caucasian (71%). Patients entered into the study on average at 29.1 (range 10–37) weeks of gestation with 57% in the third trimester, 42% in the second trimester, and 1 patient in the first trimester. Details of their previous pregnancy history are given in Table 1 which shows that the majority had had one previous pregnancy that had gone to term. 

Heartburn symptoms had commenced on average at 21.1 (SD 8.1, range 4–36) weeks of gestation with the majority starting in the second trimester (61%) and 19% commencing symptoms in both the first and third trimesters. From a total of 627 study and routine antenatal visits, heartburn symptoms had been present in the preceding 72 hours on 93% of occasions.

### 3.2. Efficacy Data

**Figure 1:**
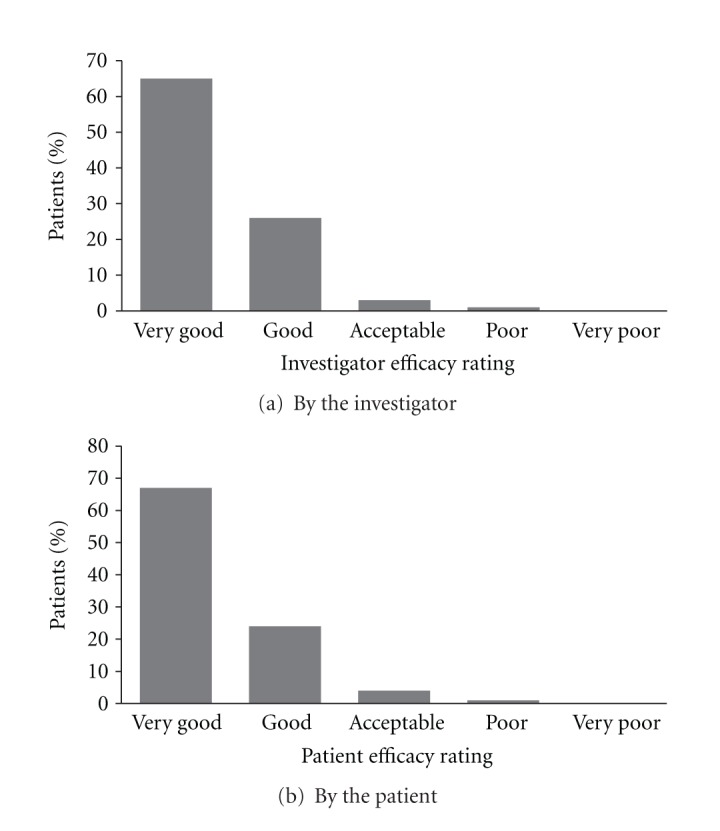
Frequency distribution for the ITT population of impression of treatment of heartburn in pregnancy by Liquid Gaviscon at study assessment visit.

For the ITT population the investigator deemed treatment to be a success (rated good or very good) in 91% of patients (95% CI 85.0–95.3%), and the breakdown of treatment impression is shown in Figure 1(a). Similarly, 90% (95% CI 84.1–94.8%) of patients claimed treatment success with LG with breakdown of responses shown in Figure 1(b). For the EE population treatment success was seen in 96% of patients (95% CI 89.8–98.9%) when assessed by the investigator and 95% (95% CI 88.4–98.3%) when assessed by the patient.

**Figure 2:**
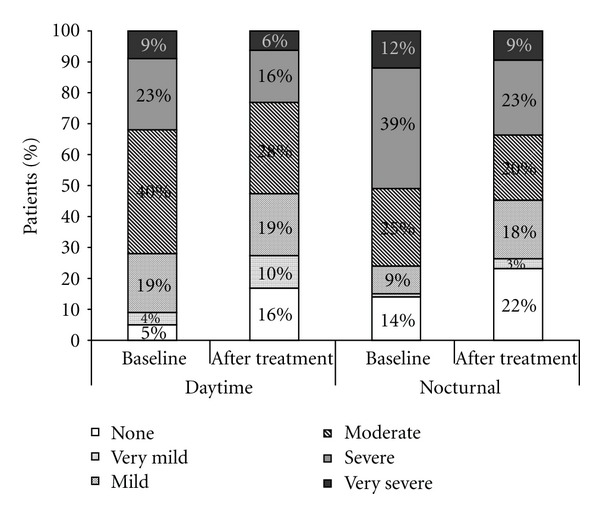
Severity of daytime and nocturnal heartburn experienced by pregnant women at baseline and after prn treatment with Liquid Gaviscon for 4 weeks. (ITT).

Interestingly, when symptom frequency and severity were broken down by daytime or nighttime, it was shown that heartburn was classified most often as severe during the night but only moderate during the day in pregnant women. At baseline, 51% of women documented severe or very severe nocturnal heartburn and this was reduced to 32% after 4 weeks of treatment. A similar extent of improvement was seen with daytime heartburn from 32% experiencing severe or very severe symptoms at baseline to 22% after 4 weeks of treatment.  Figure 2 indicates how treatment increases the proportion of patients experiencing none or milder symptoms but reduces those at the more severe end of the spectrum. The majority of patients documented relief within 10 minutes of taking the medication (67%) and most within 20 minutes (91%).

Patients took a mean (SD) of 1.8 (1.1) doses of LG per day (range 0.2–5.5), and the most common dosage volume was 20 mL (37%). The estimated daily dose per patient was 34.6 mL (SD 29.7 mL). 

Seventy-eight percent of patients continued to use the medication after the 4-week assessment period. The mean (SD) time of exposure to LG was 8.8 (5.7) weeks with the maximum being 25 weeks of use of LG. 

### 3.3. Safety Evaluation

A total of 130 births were delivered to 129 mothers (1 set of twins) with no data for 17 women. There were three intrauterine deaths (still births), and 1 baby (born prematurely) died after the post-partum visit that all occurred in South Africa. The majority of births (95%) were delivered between 36 and 44 weeks with 5% premature. APGAR scores recorded after a median time of 1 minute ranged from 2 and 10 (mode 9) and was ≥7 in 92% of babies. The APGAR scores recorded a median of 5 minutes ranged from 6 and 10 (mode 10) and was ≥7 in 95% of babies.

#### 3.3.1. Serum Electrolytes

Serum potassium and sodium levels were monitored because of the sodium load of the product and the potential impact upon blood pressure or fluid retention in the pregnant population. At baseline, mean (SD) serum sodium levels were 137.7 (2.90) mmol/L and at the study assessment visit values were similar with mean (SD) 137.8 (2.78) mmol/L. Serum potassium levels did not change throughout the study with mean (SD) serum potassium 4.0 (0.37) mmol/L at both baseline and study assessment visit. There were no patients with clinically significant deviations in serum electrolytes from the reference range.

#### 3.3.2. Adverse Events—Patients

Overall, 86 patients reported 237 emergent adverse events during the study (Table 2) and were most commonly female reproductive system disorders (29%), GI system disorders (16%), or general disorders (9%). Most adverse events reported by the mothers were disorders caused or aggravated by pregnancy (e.g., Caesarean section, hypertension, and anaemia). Only 27 events (21 patients) were considered of severe intensity and only 3 events were considered possibly or probably related to the study medication, and these were single occurrences of hypertension, diarrhoea, and nausea which lead to withdrawal of these 3 patients from the study. 

Serious adverse events were experienced by 27 patients reporting 47 events. In 16 of these serious adverse events, caesarean section was classified as an adverse event, but all had an additional adverse event associated with the caesarean section.

#### 3.3.3. Adverse Events—Fetuses

There were 18 adverse events (predominantly fetal distress) reported for 17 fetuses (Table 2), and although 7 events (6 fetuses) were deemed severe, none of the events experienced by the fetuses were considered to be possibly or probably related to the study medication. 

There were 14 serious adverse events from 13 fetuses and there were 3 intra-uterine deaths (2 episodes of placental abruption and one due to intrapartum asphyxia), but none of these serious events were considered related to the study medication.

#### 3.3.4. Adverse Events—Neonates

In 19 babies, 27 events were reported (Table 2) and 4 events (3 babies) were severe, but none were considered possibly or probably related to the study medication. 

There were 6 serious adverse events (5 neonates) with one neonatal death as a result of a premature delivery (31.5 weeks gestation) and respiratory disorders (hyaline membrane disease and bronchopleural fistula). However, none of the serious adverse events were considered possibly or probably related to the study medication.

#### 3.3.5. Safety Summary

Very few adverse events or serious adverse events were reported for mothers in the study that were considered to be related to the medication. There were no concerns over the sodium content of the product. Adverse events and fetal/neonatal deaths were consistent with the population incidences in the UK and South Africa (Table 3) and indicated no safety concerns for LG use during pregnancy. It should be noted that the infant mortality rate (perinatal and neonatal) in South Africa is variable depending on the socioeconomic status [22, 23]. Caesarean section rates are high in South Africa, but as the study group consisted of some private clinics, the rate of Caesarean section is estimated to be 30%. 

## 4. Conclusion

The prevalence of heartburn during pregnancy is high, and treatment needs to be safe to both the mother and unborn child. LG has a non-systemic mode of action by forming an alginate raft on the stomach contents to physically prevent reflux into the oesophagus [24]. Its mode of action should not pose any safety concerns to the mother or child, nor should the mode of action be affected by physiological changes seen in pregnancy. Therefore, despite its extensive use throughout pregnancy, there was a requirement to undertake a formal safety study. 

This prospective clinical study evaluated the safety and efficacy of LG for the treatment of heartburn in pregnancy in an open-label format. This study used a standard real-life dosage regimen in that patients could take medication as required (up to a maximum of 80 mL per day). Although the formal efficacy study lasted for 4 weeks, the protocol allowed patients to continue treatment if desired (of which the majority did), thus this more accurately reflected normal clinical practice and allowed assessment of longer term safety of the medication rather than a fixed short-term study.

The efficacy of LG in the treatment of heartburn in pregnancy was assessed by both investigator and patient at 4 weeks. Treatment success (good or very good response) was seen in 91% of patients as judged by the investigator and 90% from self-appraisal. This shows that LG is highly efficacious in this population and equivalent to patient perception of treatment with Gaviscon in pregnant women in the retrospective studies by Hutt et al. [7] (98%) and Uzan et al. [9] (72%). Data was comparable to the use of Gaviscon Advance in pregnant women (88%) [6] and Gaviscon Advance in the normal population (84%) [25].

When symptom frequency and severity were broken down, it showed that heartburn was classified most often as severe during the night but only moderate during the day in pregnant women. Nocturnal reflux symptoms were improved by treatment with LG. Since nocturnal symptoms are particularly important to the quality of life of pregnant women, this improvement in symptoms with LG treatment is a positive outcome.

There were no safety concerns identified from this study which evaluated LG use by pregnant women. Although the formal efficacy study was carried out over 4 weeks, the protocol allowed women to continue LG use through pregnancy if they wished, and thus the safety study was carried out with long-term real-life use. The study was carried out in the UK and South Africa, and adverse events for the mother, fetus, and baby were consistent with the population norms for those countries. Crucially, there were no disturbances in serum sodium levels as a result of the sodium load of the product. This is important in pregnant women as hypertension, preeclampsia, and oedema are important complications in pregnancy, so there were no safety concerns of LG in that respect. 

LG is licensed for use in pregnancy to treat heartburn and reflux symptoms that are common in this population, although transient can have a large impact in quality of life. Treatment should first take the form of diet and lifestyle advice followed by non-systemic therapy (antacids or alginate suspensions). The efficacy and the safety of alginate suspension in pregnancy have been established in this study and also previously [6] such that, when lifestyle improvements do not help, alginates may be the first treatment choice in this sensitive population. However, randomised controlled trials are advocated to strengthen the evidence regarding the efficacy and safety of LG.

This prospective open-label study in a large number of pregnant women in the UK and South Africa suggests that Liquid Gaviscon, 10–20 mL as required, is both safe and highly efficacious in the treatment of heartburn and GER symptoms that are prevalent and bothersome throughout pregnancy. There were no safety concerns of Liquid Gaviscon use for the mother or unborn child, and importantly there were no concerns regarding serum sodium levels. 

## Figures and Tables

**Table 1:** Previous pregnancy details of patients (safety population).

Number of patients	142
Number of previous pregnancies	
Range	0–9
0	36 (25%)
1	61 (43%)
2	21 (15%)
3 or more	24 (17%)

Number of previous pregnancies to term	
Range	0–8
0	52 (37%)
1	58 (41%)
2	22 (15%)
3 or more	10 (7%)

Number of previous pregnancies not to term	
Range	0–4
0	107 (75%)
1	26 (18%)
2	5 (4%)
3 or more	4 (3%)

**Table 2:** Breakdown of all emergent adverse events by body system. Data are number of subjects reporting event (number of events).

Body system disorder	Mother *n* = 142	Fetus *n* = 133	Baby *n* = 130
Reproductive, female	47 (68)	3 (3)	
Gastrointestinal system	25 (37)		7 (7)
Body as a whole—general	20 (22)		
Resistance mechanism	15 (19)		2 (2)
Cardiovascular—general	11 (14)		
Urinary system	12 (14)		
Central/peripheral nervous	12 (13)	1 (1)	
Red blood cell	13 (13)		
Respiratory system	9 (11)		4 (5)
Skin and appendages	6 (7)		2 (2)
Psychiatric	6 (7)		
Metabolic and nutritional	3 (4)		
Musculoskeletal system	2 (2)		1 (1)
Hearing and vestibular	2 (2)		
Vascular (extracardiac)	2 (2)		
Vision	1 (1)		1 (1)
Platelet, bleeding, and clotting	1 (1)		
Foetal		12 (12)	
Neonatal and infancy		2 (2)	7 (8)
Reproductive, male			1 (1)

Total	**86 (237)**	**17 (18)**	**19 (27)**

**Table 3:** Study and normative population data for incidences of adverse events during pregnancy.

Adverse event	UK		South Africa	
	Study	Population	Study	Population
Perinatal mortality	0	8/1000	45/1000	32.5/1000
Maternal mortality	0	12.2/100000	0	11/100000
Hypertensive disease	9%	10%	8%	10%
Eclampsia	0	1/2000	0	1/1300
Antepartum haemorrhage	3.8%	2–5%	3.4%	2–5%
Abruptio placenta	0	0.5–1.8%	2.2%	0.6%
Preterm labour	3.8%	5.1%	5.6%	11%
Caesarean section rate	26%	22%	35%	12%
Assisted vaginal deliveries	23%	10.5%	8%	5%

References from internal report by S. W. Lindow [10–21].

## Abbreviations

AGPAR: American Paediatric Gross Assessment Record
CI: Confidence interval
EE: Efficacy evaluable
FDA: Food and Drug Administration
GER: Gastrooesophageal reflux
ITT: Intention to treat
LG: Liquid Gaviscon
Prn: As required
SD: Standard deviation
GI: Gastrointestinal
CNS: Central nervous system.
